# Editorial: Innate Immunity Programming and Memory in Resolving and Non-Resolving Inflammation

**DOI:** 10.3389/fimmu.2020.00177

**Published:** 2020-02-11

**Authors:** Liwu Li, Charles McCall, Xiaoyu Hu

**Affiliations:** ^1^Department of Biological Sciences, Virginia Tech, Blacksburg, VA, United States; ^2^Department of Medicine, Wake Forest University School of Medicine, Winston-Salem, NC, United States; ^3^Institute for Immunology and School of Medicine, Tsinghua University, Beijing, China

**Keywords:** innate immune memory, inflammation dynamics, resolving and non-resolving inflammation, acute disease, chronic disease

Emerging studies reveal that “memory” responses of innate leukocytes generate adaptive reprogramming following challenges with signals of varying strength and durations. As a result, there are dynamic memory states such as priming, tolerance and exhaustion, as previously published by the Li research group in the *Frontiers of Immunology* (1). Diverse signals including microbial cues, cytokines, and metabolic products induce the varied reprogramming of inflammation and immunity, leading to distinct nature of the memory states of innate leukocytes. Innate memory dynamics profoundly impact, not only on our fundamental understanding of the immune system's cohesive functions, but may translate to interventions for infectious and inflammatory diseases, ranging from acute sepsis to chronic atherosclerosis (2–4).

To heed the emerging challenge of further defining the novel memory dynamics of innate immunity, we assembled research articles that represent various aspects of innate immune memory and are relevant to inflammatory diseases within this Research Topic. In addition to bacterial endotoxin, Walachowski et al. demonstrated that β-Glucan (BG) primes a short-term murine macrophage memory state coupled to enhanced inflammatory response to LPS, potentially by inducing Dectin-1. Dectin-1 increased expression of GM-CSF as an amplifying signal for macrophage priming induced by β-Glucan (Walachowski et al.). Shi et al. showed that myeloid cell innate immune training responses not only occur at the mature cellular stage, but during hematopoiesis. Acting at the translational level, trained immunity through BG or Bacillus Calmette-Guérin (BCG) signaling could potentially enhance host anti-microbial defenses, as reviewed by Arts et al. On a precautionary note, BG or BCG mediated immune modulation may also exacerbate inflammatory diseases, and de Bree et al. reported that the protective effects of BCG training may be limited and do not apply to protection against influenza infection. In addition to microbial products, host secondary metabolites may similarly imprint innate immune memory, such as S100A9, which Dai et al. identified as a potentiator of myeloid derived suppressive cells (MDSC) during late stage sepsis.

Molecular mechanisms dynamically reprogram innate immunity using complex and multi-layered signaling networks that are dose dependent and coupled with transient signals as well as prolonged epigenetic modifications. Intra-cellular organelle communications among mitochondria, autophagosomes, and lysosomes modulate innate immune cell decision-making (5, 6). Mitochondria controls over energy, anabolism and catabolism and cell redox state, and new evidence supports Krebs cycle metabolites as signaling molecules during the training of innate immunity and inflammation (Williams and O'Neill.). Tao et al. reported that mitochondria-localized Sirtuin 4 can resolve monocyte tolerance through rebalancing glycolysis and restoring glucose oxidation. This homeostasis promoting response suggests promise for a novel molecular targeting route to treating sepsis-associated immunoparalysis. Babaev et al. demonstrated that depletion of a mTOR complex component Rictor improves inflammation resolution in monocytes and macrophages, another possible route for chronic inflammatory reprogramming in atherosclerosis.

This collection of publications clarifies some of the mechanisms and provide a more comprehensive understanding of innate immune memory dynamics. They emphasize that future inter-disciplinary studies with integrated experimental and computational approaches at single cell level are needed. Single cell analyses during early, the post-acute, and chronic innate immune cell reprogramming among distinct tissue niches will clarify “real-life” dynamics of innate immune non-autonomous and autonomous signals, and hopefully hasten translational research of infectious and inflammatory diseases in the near future.

## Author Contributions

LL wrote the manuscript. CM and XH provided critical revision.

### Conflict of Interest

The authors declare that the research was conducted in the absence of any commercial or financial relationships that could be construed as a potential conflict of interest.
